# Drug resistance markers in *Plasmodium vivax* isolates from a Kanchanaburi province, Thailand between January to May 2023

**DOI:** 10.1371/journal.pone.0304337

**Published:** 2024-07-05

**Authors:** Thanawat Sridapan, Paweesuda Rattanakoch, Kaewkanha Kijprasong, Suttipat Srisutham

**Affiliations:** 1 Department of Clinical Microscopy, Faculty of Allied Health Sciences, Chulalongkorn University, Bangkok, Thailand; 2 Satan Prabaramee Hospital, Naung Proe, Kanchanaburi, Thailand; University of Applied Sciences Hochschule Mittweida, GERMANY

## Abstract

**Background:**

*Plasmodium vivax* has become the predominant species in the border regions of Thailand. The emergence and spread of antimalarial drug resistance in *P*. *vivax* is one of the significant challenges for malaria control. Continuous surveillance of drug resistance is therefore necessary for monitoring the development of drug resistance in the region. This study aims to investigate the prevalence of the mutation in the *P*. *vivax* multidrug resistant 1 (*Pvmdr1*), dihydrofolate reductase (*Pvdhfr*), and dihydropteroate synthetase (*Pvdhps*) genes conferred resistance to chloroquine (CQ), pyrimethamine (P) and sulfadoxine (S), respectively.

**Method:**

100 *P*. *vivax* isolates were obtained between January to May 2023 from a Kanchanaburi province, western Thailand. Nucleotide sequences of *Pvmdr1*, *Pvdhfr*, and *Pvdhps* genes were amplified and sequenced. The frequency of single nucleotide polymorphisms (SNPs)-haplotypes of drug-resistant alleles was assessed. The linkage disequilibrium (LD) tests were also analyzed.

**Results:**

In *Pvmdr1*, T958M, Y976F, and F1076L, mutations were detected in 100%, 21%, and 23% of the isolates, respectively. In *Pvdhfr*, the quadruple mutant allele (**I**_**57**_**R**_**58**_**M**_**61**_**T**_**117**_) prevailed in 84% of the samples, followed by (**L**_**57**_**R**_**58**_**M**_**61**_**T**_**117**_) in 11%. For *Pvdhps*, the double mutant allele (**G**_**383**_**G**_**553**_) was detected (48%), followed by the triple mutant allele (**G**_**383**_**M**_**512**_**G**_**553**_) (47%) of the isolates. The most prevalent combination of *Pvdhfr* (**I**_**57**_**R**_**58**_**M**_**61**_**T**_**117**_) and *Pvdhps* (**G**_**383**_**G**_**553**_) alleles was sextuple mutated haplotypes (48%). For LD analysis, the association in the SNPs pairs was found between the intragenic and intergenic regions of the *Pvdhfr* and *Pvdhps* genes.

**Conclusion:**

The study has recently updated the high prevalence of three gene mutations associated with CQ and SP resistance. Genetic monitoring is therefore important to intensify in the regions to further assess the spread of drug resistant. Our data also provide evidence on the distribution of drug resistance for the early warning system, thereby threatening *P*. *vivax* malaria treatment policy decisions at the national level.

## Introduction

Malaria remains a significant cause of parasitic public health problems in many tropical and sub-tropical countries. In 2021, 247 million cases and 619,000 malaria deaths occurred, according to a recent report by the World Health Organization (WHO) [1]. Malaria cases and deaths in Thailand continuously decreased by 90% from 2000 to 2019 [2, 3]. However, persistent transmission has remained a major public health challenge and ongoing importation, particularly in vulnerable forests and forest fringes along the international borders of Thailand [2, 3]. *Plasmodium vivax* (92.19%) is the most widespread species, accounting for most diagnosed human malaria infections in Thailand after *Plasmodium falciparum* (4.87%) [4]. Even though vivax infections are rarely mortal, it can cause severe illness in humans [5, 6].

The Thai Government has endorsed plans to achieve malaria elimination by 2024 [7]. However, widespread resistance to antimalarial drugs still poses serious challenges to accomplishing this goal [8]. The prevalence of *Plasmodium* parasite resistance toward antimalarial drugs remains high in border areas due to population migration across the borders. Three-day chloroquine (CQ) co‐administered with fourteen-day primaquine (PQ) remains the first-line treatment for all *P*. *vivax* cases for several decades in Thailand, while piperaquine (PPQ) or artemisinin combination therapies (ACTs) plus PQ were the second line treatment in case of CQ resistance (CQR) [9, 10]. There are increasing reports of treatment failure after the administration of CQ in different regions worldwide [11]. In Thailand, most *P*. *vivax* isolates are still sensitive and effectively respond well to the CQ standard regimen [12] even though there was a clinical report of CQR to *P*. vivax in a Thai pregnant woman [13]. This evidence indicates that CQR was already present in Thailand. Meanwhile, sulfadoxine‐pyrimethamine (SP), which is a combination anti-malarial drug was used in mixed infection cases or at the areas with suspected CQR in falciparum malaria in Thailand during 1972–1982. SP was also one of the partner drugs for the treatment of uncomplicated falciparum malaria and intermittent presumptive treatment (IPT) for infants, children, and pregnant women [14, 15]. In Thailand, SP has never been used as a first‐line treatment for vivax malaria [10]. During intensive SP use for the treatment of *P*. *falciparum* through coinfection with *P*. *vivax* in endemic areas [16, 17], together with the practice of malaria treatment without microscopic confirmation, this evidence leads to *P*. *vivax* being exposed to SP selective drug pressure inadvertently, resulting in the emergence of drug resistance to SP in *P*. *vivax* populations [18]. Resistance of the *P*. *vivax* to SP was highly reported along the Thai–Myanmar and Thai–Cambodia borders [19–21]. Self-treatment of fever or proper use of SP, including co-infection with *P*. *vivax* and *P*. *falciparum* in those endemic areas, leads to highly continue SP selection pressure onto *P*. *vivax* [16, 21].

Several molecular markers that show an association with drug resistance in *P*. *vivax* isolates have been identified [22]. *P*. *vivax* multidrug resistance (*Pvmdr1*), homologous to *P*. *falciparum* orthologs (*Pfmdr1*), has been reported to be correlated with CQ resistance [23, 24]. *Pvmdr1* gene encodes the digestive vacuole (DV) transmembrane proteins, which function as a CQ transporter [25]. When *Pvmdr1* was mutated, CQ diffusion into the DV of the parasite was reduced, conferring CQR [22]. However, the role of *Pvmdr1* in CQR remains unclear [26]. The Y976F mutation in *Pvmdr1* is associated with decreased *in vitro* sensitivity to CQ [23, 27]. A point mutation in Y976F and F1076L has been reported as an association with the clinical response of vivax malaria to CQR [13]. These two mutations were also used as molecular markers for monitoring the occurrence and spread of *P*. *vivax* CQR from many malaria-endemic regions [20, 28–30]. Meanwhile, dihydropteroate synthase (DHPS) and dihydrofolate reductase (DHFR) are enzymes in the folate biosynthesis pathway of the malarial parasite that are drug targeted of S and P, respectively [31]. SP resistance in *P*. *vivax* is caused by point mutations in antifolate resistance genes *Pvdhps* and *Pvdhfr* [22]. Mutation in these two genes causes alterations of amino acid residues in the enzyme-binding pockets leading to reduce the affinity of the enzyme for the drug [32]. Molecular studies have identified specific point mutations in the *Pvdhfr* gene in codons F57I/L, S58R, T61M, and S117T/N, while three mutations in codons S382A/C, A383G, and A553G/C have been detected in the *Pvdhps* gene [16, 24, 33, 34]. Point mutations of these genes are widespread in *P*. *vivax* populations worldwide [19–21, 35–39], resulting in an altered clinical response to SP [40]. The presence of mutations associated with CQ and SP resistance in *P*. *vivax* was previously reported in the Thai–Myanmar border regions [19–21, 29]. In response to this situation, continuous monitoring of the emergence and spread of antimalarial drug resistance is required to investigate the current status of the CQ and SP drug resistance in *P*. *vivax* cases in this region. The data provided is important for Thailand’s existing national malaria control strategy.

The aim of this study was to investigate the prevalence of genes potentially linked to drug resistance, including *Pvmdr1*, *Pvdhfr*, and *Pvdhps*, in *P*. *vivax* parasites isolated from Kanchanaburi, a province in western Thailand bordering Myanmar between January and May 2023.

## Materials and methods

### Study site

The study was conducted at the Satan Prabaramee Hospital, Naung Proe, Kanchanaburi province, Thailand, during January and May 2023. Kanchanaburi province (14° 1’-10" N; 99° 31’-52" E) is located in the west of Thailand. The province is mountainous, and its climate is defined as tropical, with a monsoon season from May to October and a dry season from November to April. The temperature typically varies from 20 to 37°C. This region was selected as the study site because it was a malarial endemic area where highly mobile populations can contribute to cross-border transmission along the Thai-Myanmar border frequently [41]. Kanchanaburi is one of the top three provinces for malaria cases in Thailand, according to reports from the Department of Disease Control, Ministry of Public Health, Thailand [42]. The inclusion criterion was adults (aged 20 years and over) presenting malaria infections, whereas the exclusion criterion was non-consent.

### Ethical considerations

The study protocol was reviewed and approved by the Institutional Review Board for human research of Chulalongkorn University (Permit number: COA 010/66), Bangkok, Thailand. All adult participants were given a detailed explanation of study protocols and procedures. All methods were carried out in accordance with the Declaration of Helsinki and regulations prescribed by the above organizations. Written or thumbprint informed consent was obtained for the use of remnant blood samples from study participants.

### Samples

The sample size was calculated based on a previous prevalence of *P*. *vivax* infection in western Thailand of 3.8% [43] at a confidence interval (CI) of 99% and a margin of error at 5%, using a single population proportion formula: *n* = [(Z_α/2_)^2^p(1-p)] / d^2^ = [(2.576)^2^0.038(1–0.038)] / 0.05^2^ = 98, where *n*  =  sample size, (Z_α/2_)^2^ =   a confidence interval (CI) of 99%, p  =  prevalence of malaria and d  =  margin of error (5%). This gives a sample size of 98. Thus, a total of 100 participants were recruited for this study. The samples were obtained from 100 confirmed *P*. *vivax*-infected patients from January to May 2023. All patients presenting with malaria-related signs were diagnosed and reported by the Satan Prabaramee Hospital in a Kanchanaburi province. After venous blood collection was performed, thin blood films were prepared at the time of sample, stained with Giemsa’s staining and examined under the light microscope by two independents experienced microscopists in the identification of malaria parasites. A blood smear was interpreted as negative results if no parasites were observed under at least 100 high-power fields. The parasitemia was calculated as the formula: [(counted parasites / counted leucocytes) × the assumed number of leucocytes per microliter of blood (8 × 10^3^) [44].

### DNA extraction

Genomic DNA was extracted from 200 μl of the remainder of the whole blood samples using QIAamp DNA Blood Kits (Qiagen, Germany) according to the manufacturer’s instructions. The DNA elution volume was 200 μl in elution buffer, as provided by kits. The extracted DNA was stored at -20°C until used.

### Nested PCR

A nested PCR previously described [45–47] for amplifying the 18S ribosomal RNA (rRNA) gene of *Plasmodium* spp. was used to confirm the *P*. *vivax* parasite, with some modifications: (i) using 1.5 mM of MgCl_2_, and (ii) changing the annealing step to 30 cycles at 64°C. The primer sequences and reaction conditions were performed in the Table 1 [45]. The reactions were carried out in a Mastercycler EP Gradient S (Eppendorf, Germany) in a total volume of 20 μl containing 1× PCR buffer, 1.5 mM MgCl_2_, 1 U *Taq* DNA polymerase (Vivantis, Malaysia), 0.2 mM dNTPs (Biotechrabbit, Germany), 0.25 μM each of forward and reward primers (Bio Basic Inc., Canada), and 2 μl of DNA template. One microliter of the nest-1 products was used as a template for the second round of PCR (nest-2) using the *P*. *vivax* species-specific primers (Table 1) [47]. Nest-2 PCR products were electrophoresed separately on a 1.5% agarose gel (SERVA Electrophoresis GmbH, Germany), stained with SERVA DNA Stain G (SERVA Electrophoresis GmbH, Germany), and visualized under UV light using a UV transilluminator (Bio-Rad, USA) to visualize and size the bands. Reaction with no DNA template having sterile distilled water was used as blank control.

**Table 1:** Nucleotide primers and PCR conditions for identification of *Plasmodium vivax* and drug resistance marker genes.

Target	PCR cycles	Primer sequences (5’-3’)	PCR conditions	Size (bp)	Reference
*P*. *vivax*	Nest-1 (rPLU1)	F-TCAAAgATTAAgCCATgCAAgTgA	94°C, 5 min [94°C 30 s, 58°C 30 s, 72°C 45 s] × 25 cycles, 72°C, 7 min	1,700	[45]
	Nest-1 (rPLU5)	R-CCTgTTgTTgCCTTAAACTTC			
	Nest-2 (rVIV1)	F-CgCTTCTAgCTTAATCCACATAACTgATAC	94°C, 5 min [94°C 30 s, 64°C 30 s, 72°C 45 s] × 30 cycles, 72°C, 7 min	120	[46]
	Nest-2 (rVIV2)	R-ACTTCCAAgCCgAAgCAAAgAAAgTCCTTA			
*Pvmdr1*	Nest-1	F- CACCgCACCAgTTgATTCCT	94°C, 5 min [94°C 30 s, 62°C 30 s, 72°C 3 min] × 25 cycles, 72°C, 7 min	2,804	[48]
		R- CTTATATACgCCgTCCTgCAC			
	Nest-2	F- ggATAgTCATgCCCCAggATTg	94°C, 2 min [94°C 30 s, 62°C 30 s, 72°C 30 s] × 30 cycles, 72°C, 7 min	604	[49]
		R- CATCAACTTCCCggCgTAgC			
*Pvdhfr*	Nest-1	F- CACCgCACCAgTTgATTCCT	94°C, 5 min [94°C 30 s, 56°C 30 s, 72°C 1 min] × 20 cycles, 72°C, 7 min	983	[50]
		R- CCTCggCgTTgTTCTTCT			
	Nest-2	F- CCCCACCACATAACgAAg	94°C, 2 min [94°C 30 s, 56°C 30 s, 72°C 30 s] × 25 cycles, 72°C, 7 min	755	
		R- CCCCACCTTgCTgTAAACC			
*Pvdhps*	Nest-1	F- gATggCggTTTATTTgTCg	94°C, 5 min [94°C 30 s, 56°C 30 s, 72°C 1 min] × 20 cycles, 72°C, 7 min	1,009	[50]
		R- gCTgATCTTTgTCTTgACg			
	Nest-2	F- gCTgTggAgAggATgTTC	94°C, 2 min [94°C 30 s, 56°C 30 s, 72°C 30 s] × 25 cycles, 72°C, 7 min	731	
		R- CCgCTCATCAgTCTgCAC			

### PCR amplification and sequencing

Partial regions flanking major mutations of drug resistance marker genes, including *Pvmdr1*, *Pvdhfr*, and *Pvdhps* were amplified by nested PCR using primer sets from previous studies (Table 1) [48–50]. The reactions were performed in a total volume of 20 μl containing 1× PCR buffer, 1.5 mM MgCl_2_, 1 U *Taq* DNA polymerase (Vivantis, Malaysia), 0.2 mM dNTPs (Biotechrabbit, Germany), 0.2 μM each of forward and reward primers (Bio Basic Inc., Canada), and 2 μl of DNA template. The PCR conditions were carried out in Table 1. The nest-2 PCR products were analyzed, as described above. The PCR products were then purified using kits (Bio-Helix, Taiwan) and sent for Sanger DNA sequencing to Macrogen using the ABI 3730 XL DNA Analyzer (South Korea).

### Sequence polymorphism and statistical analyses

Sanger sequences generated in this study (*n* = 100) were analyzed using the free software Bioedit version 7.2.5. Low-quality scores from sequencing errors on nearby upstream bases were cropped.

A lot of noise or background along the bottom of the trace was re-analyzed. All overlapping peaks in electropherograms (heterozygous) were also considered. Sanger sequences whose electropherograms showed single peaks were involved in the analysis. The sequenced fragments were validated by BLASTN searches (https://blast.ncbi.nlm.nih.gov/Blast.cgi). Consensus sequences of all three drug resistance marker genes were then aligned using the clustalW algorithm, as viewed in Bioedit software to identify polymorphisms compared with the following reference sequences of *P*. *vivax*; *Pvmdr1* (accession no. XM001613678), *Pvdhfr* (accession no. XM001615032), and *Pvdhps* (accession no. XM001617159). Each antimalarial drug resistance gene’s nucleotide and deduced amino acid sequences were analyzed and compared to reported resistance-associated mutations. Allele frequencies were calculated as percentages (number of mutants / total number of tested samples). The comparison of haplotype frequency in each gene population was also calculated. Chi square and 2-tailed Fisher’s exact tests were used to determine the difference in prevalence of mutant alleles of each SNP between years using SPSS (version 29.0.0.0 SPSS Inc., IL, USA) for window. Statistical significance was defined as a *P*- value < 0.001. In addition, both intergenic and intragenic linkage disequilibrium (LD) tests were performed by calculating the r^2^ values to determine the association between the SNPs of the *Pvdhfr* and *Pvdhps* genes using Haploview software [51]. The strength of the statistical significance of LD between the SNPs was represented by the darkness of the boxes.

## Results

### Analysis of SNPs in *P*. *vivax* genetic markers

**Fig 1:**
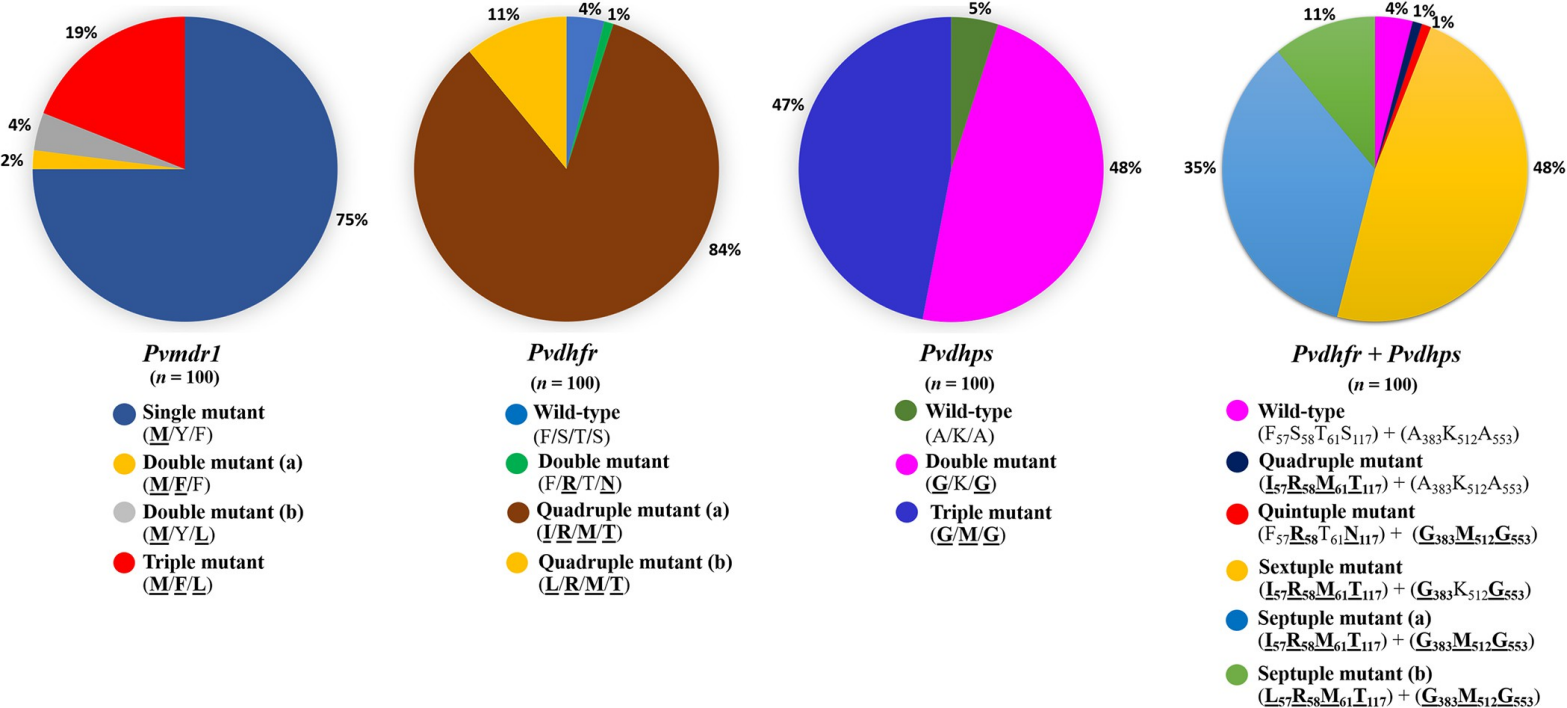
Prevalence of *Pvmdr1*, *Pvdhfr*, *Pvdhps* and *Pvdhfr + Pvdhps* haplotypes in *P*. *vivax* isolates from a Kanchanaburi province, Thailand. Pie charts show haplotype frequencies as percentages among 100 *P*. *vivax* isolates. Mutant amino acids are shown in bold with underlined letters.

A total of 100 samples infected by a single population of *P*. *vivax* were obtained in the analysis. All samples were diagnosed by microscopy. The parasitemia ranges of the participants was 800–6,640 parasites / μl. 100 microscopy-positive malarial isolates were then confirmed by nested PCR, which showed *P*. *vivax* mono-infections without a mixed infection. Genetic markers *Pvmdr1*, *Pvdhfr*, and *Pvdhps* were successfully amplified and resulted in 100 sequences (S1 File and S1 Table). A summary of the observed allelic distribution of *P*. *vivax* markers is given in Tables 2–4 and shown in Fig 1.

**Table 2:** Prevalence of mutation of the genetic markers conferring antimalarial drug resistance in *P*. *vivax* isolates from a Kanchanaburi province, Thailand collected between January to May 2023.

Gene	Codon and mutation	Allele frequency (%)
		(*n* = 100)
*Pvmdr1*	T958M	100
	Y976F	21
	F1076L	23
*Pvdhfr*	F57I	84
	F57L	11
	S58R	96
	T61M	95
	S117T	95
	S117N	1
*Pvdhps*	A383G	95
	K512M	47
	A553G	95

Allele frequencies are calculated as percentages (number of mutants / total number of tested samples)

**Table 3:** Prevalence of *Pvmdr1*, *Pvdhfr* and *Pvdhps* haplotypes in *P*. *vivax* isolates from a Kanchanaburi province, Thailand collected between January to May 2023.

Gene	Haplotype	Codon	Haplotypes frequency (%)
*Pvmdr1*		958/976/1076	(*n* = 100)
	Wild-type	T/Y/F	0
	Single mutant	**M**/Y/F	75
	Double mutant (a)	**M**/**F****/**F	2
	Double mutant (b)	**M**/Y/**L**	4
	Triple mutant	**M**/**F**/**L**	19
*Pvdhfr*		57/58/61/117	(*n* = 100)
	Wild-type	F/S/T/S	4
	Double mutant	F/**R**/T/**N**	1
	Quadruple mutant (a)	**I**/**R**/**M**/**T**	84
	Quadruple mutant (b)	**L**/**R**/**M**/**T**	11
*Pvdhps*		383/512/553	(*n* = 100)
	Wild-type	A/K/A	5
	Double mutant	**G**/K/**G**	48
	Triple mutant	**G**/**M**/**G**	47

Mutant amino acids are shown in bold with underlined letters

Haplotype frequencies are calculated as percentages (number of haplotypes / total number of tested samples)

**Table 4:** Distributions of *Pvdhfr* and *Pvdhps* combination alleles among 100 *P*. *vivax* isolates from a Kanchanaburi province, Thailand.

*Pvdhfr* allele	*Pvdhps* allele			Combination type	Haplotype frequency (%) (*n* = 100)
	A_383_	K_512_	A_553_		
Wild type (F_57_S_58_T_61_S_117_)	A	K	A	Wild	4
Double mutant (F_57_**R**_**58**_T_61_**N**_**117**_)	**G**	**M**	**G**	Quintuple	1
Quadruple mutant (a) (**I**_**57**_**R**_**58**_**M**_**61**_**T**_**117**_)	A	K	A	Quadruple	1
	**G**	K	**G**	Sextuple	48
	**G**	**M**	**G**	Septuple (a)	35
Quadruple mutant (b) (**L**_**57**_**R**_**58**_**M**_**61**_**T**_**117**_)	**G**	**M**	**G**	Septuple (b)	11

Mutant amino acids are shown in bold with underlined letters

Haplotype frequencies are calculated as percentages (number of haplotypes / total number of tested samples)

#### Pvmdr1

Three mutations at codons T958M, Y976F, and F1076L were observed in the *Pvmdr1*gene. The T958M mutation was found in all sequenced isolates (100%), while at codons Y976F and F1076L were detected with frequencies of 21% and 23%, respectively (Table 2). This gave rise to four haplotypes comprised by a single-mutant allele (**M**_**958**_) being the predominant (75%), double-mutant allele (a) (**M**_**958**_**F**_**976**_) (2%), double-mutant allele (b) (**M**_**958**_**L**_**1076**_) (4%), and triple-mutant allele (**M**_**958**_**F**_**976**_**L**_**1076**_) (19%) (Fig 1 and Table 3).

#### Pvdhfr

Mutations in the *Pvdhfr* gene at codons 57, 58, 61, and 117 were observed in 95%, 96%, 95%, and 96%, respectively (Table 2). Analysis of *Pvdhfr* haplotype revealed four distinct allelic forms, including the WT haplotype (F_57_S_58_T_61_S_117_), double-mutations (**R**_**58**_**N**_**117**_), quadruple-mutations (a) (**I**_**57**_**R**_**58**_**M**_**61**_**T**_**117**_), and quadruple mutations (b) (**L**_**57**_**R**_**58**_**M**_**61**_**T**_**117**_) in 4%, 1%, 84%, and 11%, respectively (Fig 1 and Table 3). Besides those non-synonymous polymorphisms, synonymous mutations were detected at codon Y69 (TTT→TAC) in the WT allele (S1 Table). In addition, two tandem repeat variations between amino acid positions 88 and 103 of *Pvdhfr* (Fig 4A) were detected in all isolates (S1 Table). Type 1 or wild type, i.e. three repeated sets of four amino acids (5′-GGDN-3′) was most common in isolates (99%), while type 2, which represents six deleted amino acids at codons 98–103 (5′-THGGDN-3′) were found in one isolate (1%). Type 2 isolate samples also carried double mutations at codons 58 and 117 (**R**_**58**_**N**_**117**_) (S1 Table).

#### Pvdhps

Mutations at codon 383, 512, and 553 in the *Pvdhps* gene were detected in 95%, 47%, and 95%, respectively (Table 2). Haplotype analysis of *Pvdhps* revealed three distinct allelic forms, including the WT haplotype (A_383_K_512_A_553_), double mutations (**G**_**383**_**G**_**553**_), and triple mutations (**G**_**383**_**M**_**512**_**G**_**553**_) in 5%, 48%, and 47%, respectively (Fig 1 and Table 3). There were no mutations observed at the codon S_382_ in all isolates.

#### Frequency of combined *Pvdhfr* and *Pvdhps* haplotypes

The correlations between genotypes of the *Pvdhfr* and *Pvdhps* alleles were determined among *P*. *vivax* isolates (Fig 1 and Table 4). Four *Pvdhfr* and three *Pvdhps* alleles were combined and identified as six distinct haplotypes. The most prevalent combination was a quadruple mutant (a) (**I**_**57**_**R**_**58**_**M**_**61**_**T**_**117**_) of *Pvdhfr*, combined with a double mutant allele (**G**_**383**_**G**_**553**_) of *Pvdhps–*forming sextuple mutated haplotypes (48%) of isolates, followed by a triple mutant allele (**G**_**383**_**M**_**512**_**G**_**553**_) of *Pvdhps–*forming septuple (a) mutated haplotypes (35%). A triple mutant allele (**G**_**383**_**M**_**512**_**G**_**553**_) of *Pvdhps* combined with a quadruple mutant (b) (**L**_**57**_**R**_**58**_**M**_**61**_**T**_**117**_) allele of *Pvdhfr*–forming septuple (b) mutated haplotypes was found in 11%, followed by a double mutant allele (**R**_**58**_**N**_**117**_) of *Pvdhfr*–forming quintuple mutated haplotypes in one isolate. Four of the isolates carried wild-type alleles of both genes (Fig 1 and Table 4).

#### Linkage disequilibrium (LD) analysis

**Fig 2:**
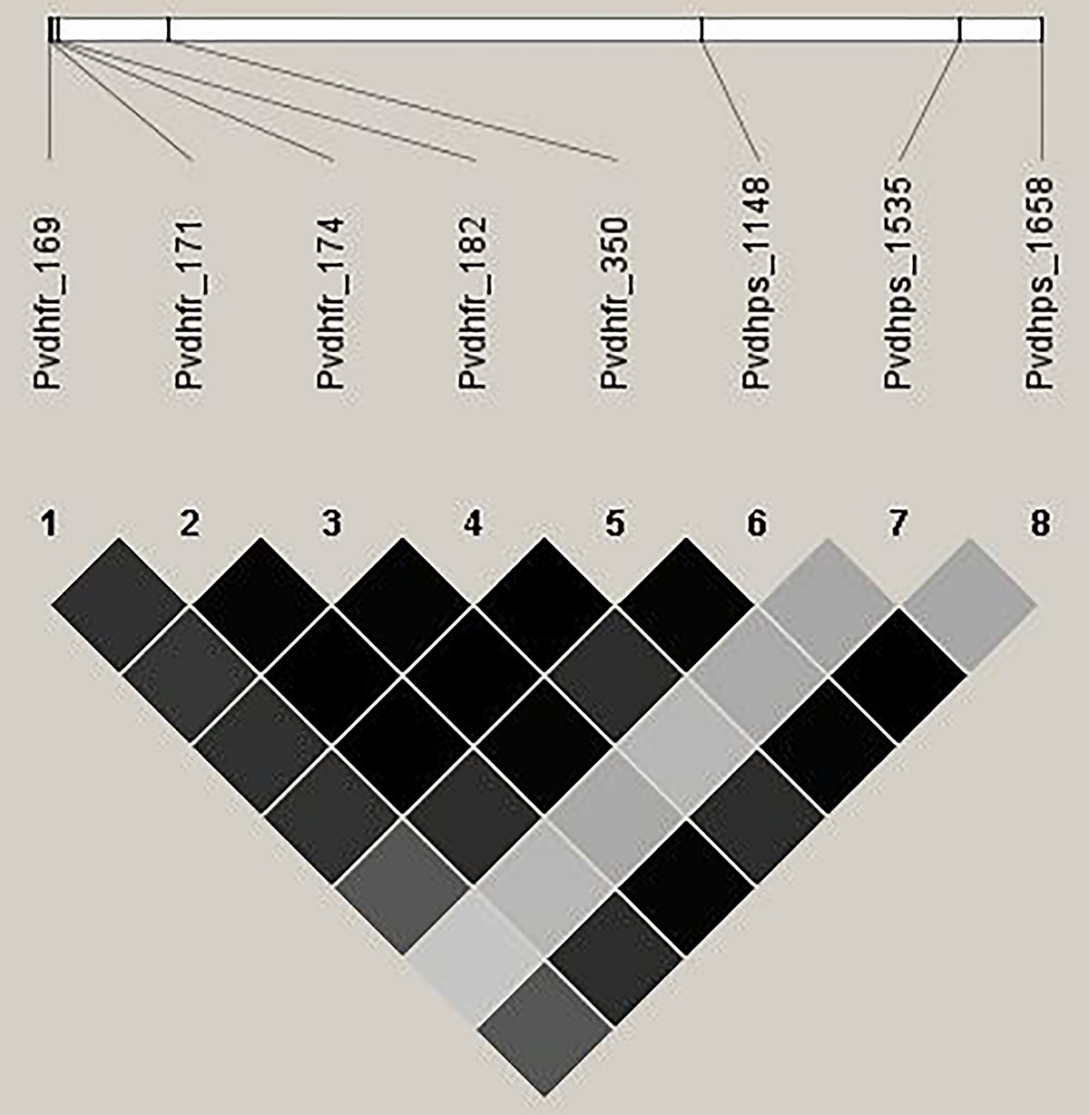
Haploview analysis for r^2^ pairwise measures of linkage disequilibrium (LD) between the SNPs pairs of *Pvdhfr* and *Pvdhps* implicated in drug resistance of *P*. *vivax* from a Kanchanaburi province, Thailand. For *Pvdhfr*, substitution mutations of T169A, C171A, C174G, C182T and G350C indicated the mutations at codons **I**_**57**_, **L**_**57**_, **R**_**58**_, **M**_**61**_ and **T**_**117**_, respectively, Meanwhile, the C1148G, A1535T and C1658G related to the mutations at codons **G**_**383**_, **M**_**512**_ and **G**_**553**_ of *Pvdhps*, respectively. The magnitude of the statistical significance of LD between the SNPs was represented by the extent of darkness in the boxes. Dark gray to black boxes correspond to pairs of SNPs with strong evidence of LD, while light gray to white indicate a lower degree of correlation.

To evaluate the LD associations of *Pvdhfr* and *Pvdhps* haplotypes, the LD pattern for each SNP in the *Pvdhfr* and *Pvdhps* genes was assessed (Fig 2). For *Pvdhfr*, substitution mutations of T169A, C171A, C174G, C182T and G350C indicated the mutations at codons **I**_**57**_, **L**_**57**_, **R**_**58**_, **M**_**61**_ and **T**_**117**_, respectively. Meanwhile, the C1148G, A1535T and C1658G related to the mutations at codons **G**_**383**_, **M**_**512**_ and **G**_**553**_ of *Pvdhps*, respectively. Several statistically significant intragenic and intergenic associations were detected among the SNPs pairs in both the *Pvdhfr* and *Pvdhps* genes.

## Discussion

Border malaria, particularly along the Thai–Myanmar border area, remains an obstacle to malaria elimination since the risk of malaria reintroduction from the migrant population still exists. Though *P*. *vivax* malaria was common along the Thailand–Myanmar border, *P*. *vivax* infections were still important causes of hospitalization. Molecular surveillance is therefore important to monitor the prevalence of drug resistance that is associated with drug efficacy in this region. This study provided up-to-date evidence on drug resistance in *P*. *vivax* at Kanchanaburi province along Thai–Myanmar border through the analysis of the polymorphisms of antimalarial drug resistance in 2023.

**Fig 3:**
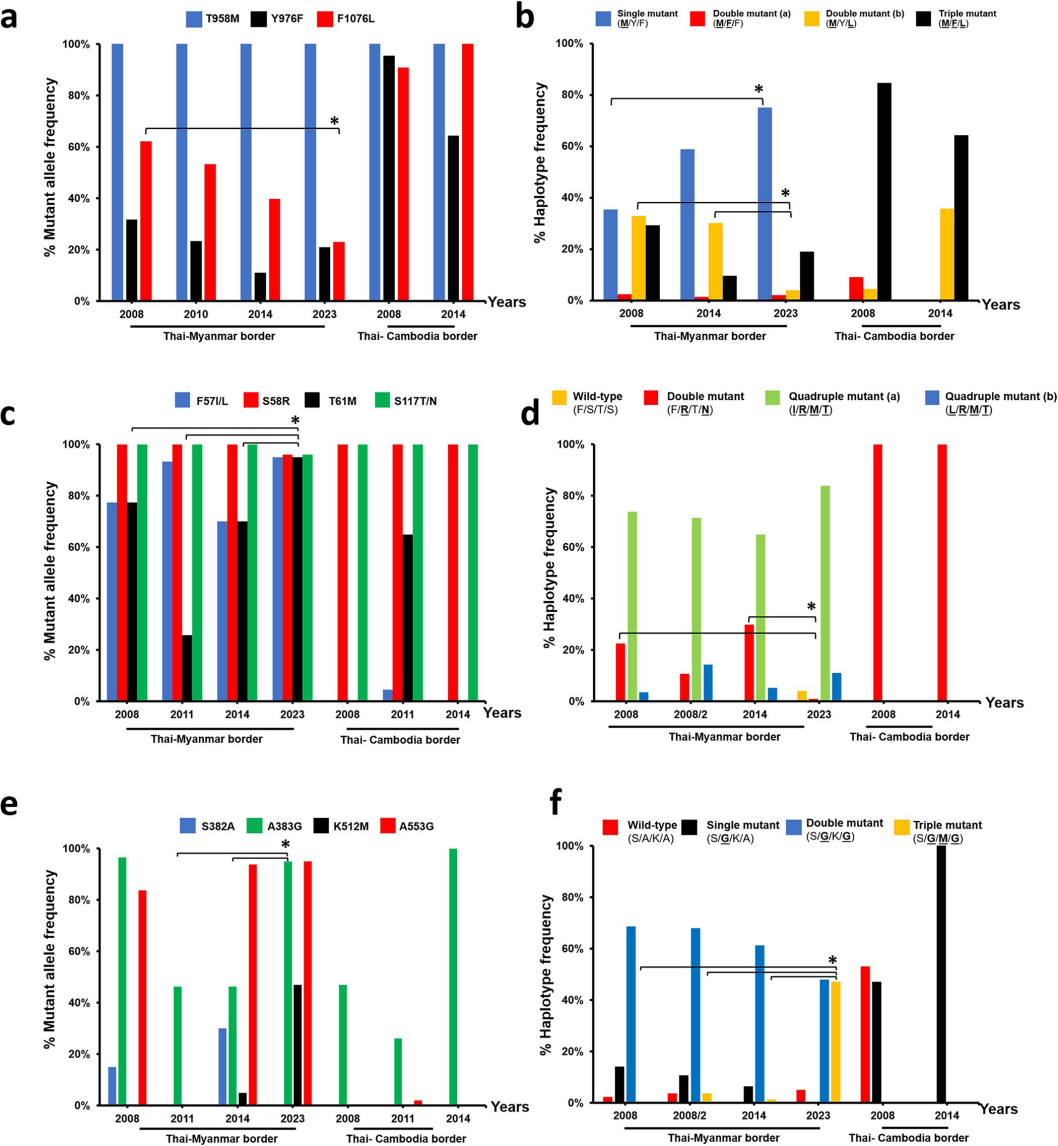
Mutant allele and haplotype frequencies of *Pvmdr1* (a, b), *Pvdhfr* (c, d), and *Pvdhps* (e, f) in *P*. *vivax* isolates from a Kanchanaburi province, western Thailand. Data were combined from previously published surveys on the Thai–Myanmar and Thai–Cambodia borders between 2008 [20], 2008/2 [19], 2010 [29], 2011 [21], 2014 [20] and 2023 in this study. Mutant allele frequencies (a, c, e) and haplotype frequencies (b, d, f) are represented as percentages on the y-axis, while the x-axis indicate time (in years). The different bar charts indicate different mutant alleles. Mutant amino acids are shown in bold with underlined letters. The difference in the mutant allele and haplotype frequencies between year was calculated by Chi square and 2-tailed Fisher’s exact test with statistically significant difference at *P*-value < 0.001.

There are increasing worldwide reports of chloroquine treatment failures of *P*. *vivax* malaria [29, 52–54]. Though new drugs are now available to replace CQ plus PQ, such as mefloquine (MQ), tafenoquine (TQ), and artemisinin-based combination therapy (ATC) [22], those drugs are more costly than CQ plus primaquine [55]. Alternative treatment regimens such as artemether-lumefantrine for treating CQR in vivax malaria have also been established and evaluated for their antimalarial activities [56]. It was shown that artemether-lumefantrine was as effective as CQ against vivax malaria [56]. These drugs may be used as the first-line drug for the treatment of vivax malaria in a mixed infection of falciparum and vivax malaria areas. However, alternative regimens are now not generally available. These alternative treatment policies have not been proposed based on clinical studies or therapeutic efficacy. Efficient methods are required to assess the potential for resistance to current standard treatments. Molecular markers have not been confirmed yet. Therefore, monitoring *P*. *vivax*’s resistance to anti-malarial drugs is still important for an earlier warning system. Unlike *P*. *falciparum*, *in vitro*, studies to assess drug resistance in *P*. *vivax* are still difficult to conduct due to the asynchronous blood stage of the parasite, including relapse, recrudescence, and reinfection of this parasite [29]. The molecular mechanisms of drug resistance in *P*. *vivax* remain ambiguous. A genetic marker of CQR in *P*. *vivax* is also still not clear. The search for CQ resistance markers in *P*. *vivax* relied on *P*. *falciparum* ortholog, namely *Pvmdr1* in *P*. *vivax* [57]. However, based on the currently available data on drug resistance markers in *P*. *vivax*, *Pvmdr1* mutations have been suggested to be candidate markers of resistance to the frontline treatment drug CQ in *P*. *vivax* [23, 58]. This study found the T985M mutation in all isolates (100%), similar to a previous report in Thailand that showed the T985M allele is dominant on the Thai–Myanmar and Thai–Cambodia borders [20, 29] (Fig 3A). This allelic variant is fixed in Asia and Africa isolates but is not responsible for CQR in *P*. *vivax* [59]. The F1076L mutation showed a prevalence of 23%. There was a decrease in the prevalence of the 1076L mutant allele on the Thai–Myanmar border with statistical significance. In contrast, this allelic variant was still found in a high prevalence on the Thai–Cambodia borders between 2008 and 2014 (Fig 3A, S2 Table) [20]. The mutation Y976F, which has been found an association with decreased susceptibility to CQ in *in vitro* studies [23, 27] was detected in 21%. This mutation is less common along the Thai-Myanmar border, similar to a previous report [20, 29, 60] compared to the Thai–Cambodia borders. Haplotype analysis showed that the single mutant (**M**_**958**_) is more frequent in this study (75%), a result congruent with previous research in the Thai–Myanmar border [20, 29] (Fig 3B). The prevalence of this haplotype has increased (35.4% in 2008 and 75% in 2023) with statistical significance (Fig 3B, S3 Table). In contrast, at the Thai–Cambodia border, the triple mutant (**M**_**958**_**F**_**976**_**L**_**1076**_) was dominant [20]. The double-mutant (a) (**M**_**958**_**F**_**976**_) and triple mutant (**M**_**958**_**F**_**976**_**L**_**1076**_) alleles that carried the Y976F mutation were detected in both borders. Notably, these two haplotypes were more prevalent along the Thai–Cambodia border than the Thai–Myanmar border (Fig 3B). Even though there are few case reports of CQR *P*. *vivax* carried the double (**F**_**976**_**L**_**1076**_) mutations in Thailand [13], CQ remains the drug of choice and an effective treatment for most *P*. *vivax* malaria cases in Thailand [12]. CQ-sensitive strains of *P*. *vivax* are mostly found in this study. In contrast, a high prevalence of CQR in *P*. *vivax* has occurred in Indonesia, Malaysia, and Papua New Guinea, which has been linked with life-threatening complications of malaria due to treatment failure. Reports of drug resistant were found in this study, indicating that resistant phenotypes would continue to spread in these regions. However, the **F**_**976**_**L**_**1076**_ mutations in clinical CQR have not been evaluated yet in patients infected with mutant *Plasmodium* species. Clinical studies at different geographic sites have reported that *Pvmdr1* mutations are not linked with CQR [61, 62]. The functional studies for *Pvmdr1* mutations also need to be fully understood. In addition, susceptibility to MQ was significantly lower in *P*. *vivax* after the introduction of MQ regimens in Cambodia during 2017–2018 [63]. The associations between susceptibility and mutation in *Pvmdr1* were not only in CQR but also in mefloquine resistance (MQR). MQ has been used against CQR and SP-resistant falciparum malaria at the Thai-Cambodian border [64]. MQR was associated with the *Pfmdr1* gene in *P*. *falciparum* [58]. Ongoing selection of decreased susceptibility to MQ in *P*. *vivax* in a neighboring country should be aware. This was evidence of a cross-resistance to other antimalarials due to *Pvmdr1* mutations. Thus, close supervision of the efficacy of CQ together with *in vitro* susceptibility tests to determine at the clinical level in order to confirm gene polymorphism-related drug resistance and therapeutic failure should encourage constant surveillance for monitoring the occurrence of CQR, and possible MQR in *P*. *vivax* in Thailand.

Due to the wide use of SP to treat mixed *P*. *falciparum* and *P*. *vivax* infections, the emergence of SP resistance in *P*. *vivax* has been reported worldwide. Mutation in the *Pvdhfr* and *Pvdhps* has been widely reported and associated with spreading SP-resistance in the *P*. *vivax* parasite in countries within the Greater Mekong Sub-region (GMS) [40]. The result in this study aggregated with previously published survey showed high level resistance of *P*. *vivax* parasite to SP at Thai–Myanmar border, which was concordant with previous reports [19–21]. For *Pvdhfr* gene, confer resistance to pyrimethamine, the mutations at codons 57, 58, 61 and 117 were the most prevalent on the Thai–Myanmar border, similar to a previous report from Thailand (Fig 3C) [19–21]. In contrast at the Thai–Cambodia border, codons 58, 61 and 117 were predominant. In this study, the prevalence of mutation frequency at codons 57, 58, 61 and 117 were very similar (95–96%), compared with a previous report on this border region [20, 21] (Fig 3C). Haplotype analysis of *Pvdhfr* revealed a high prevalence of quadruple mutant (**I****/****L**_**57**_**R**_**58**_**M**_**61**_**T**_**117**_) (95%), where is commonly prevalent along the Thai–Myanmar border (Fig 3D) [19–21, 65]. Quadruple mutant (a) (**I**_**57**_**R**_**58**_**M**_**61**_**T**_**117**_) was the most prevalent (84%) in this study, followed by quadruple mutant (b) (**L**_**57**_**R**_**58**_**M**_**61**_**T**_**117**_) (11%), similar to those reported at the same border region [19, 20] (Fig 3D). Compared to previous reports at the Thai–Myanmar border, this study found a double-mutant allele (**R**_**58**_**N**_**117**_) in very low frequency (1%) with statistical significance (Fig 3D, S3 Table). On the contrary, a very high prevalence of this allele was observed at the Thai–Cambodia border. Unlike previous reports [20, 21], wild-type alleles (F_57_S_58_T_61_S_117_) were detected (4%) in this study. This allele carried non-synonymous mutations at codon Y69 (TTT→TAC). Double-mutant allele (**R**_**58**_**N**_**117**_) and quadruple-mutant allele (**I****/****L**_**57**_**R**_**58**_**M**_**61**_**T**_**117**_) are likely associated with reducing their susceptibility to pyrimethamine [33, 66, 67]. The high prevalence of these mutant alleles in this study agreed with the report of other previous studies [19–21, 68]. Our finding indicated that pyrimethamine resistance is still high over time on the Thai–Myanmar border.

**Fig 4:**
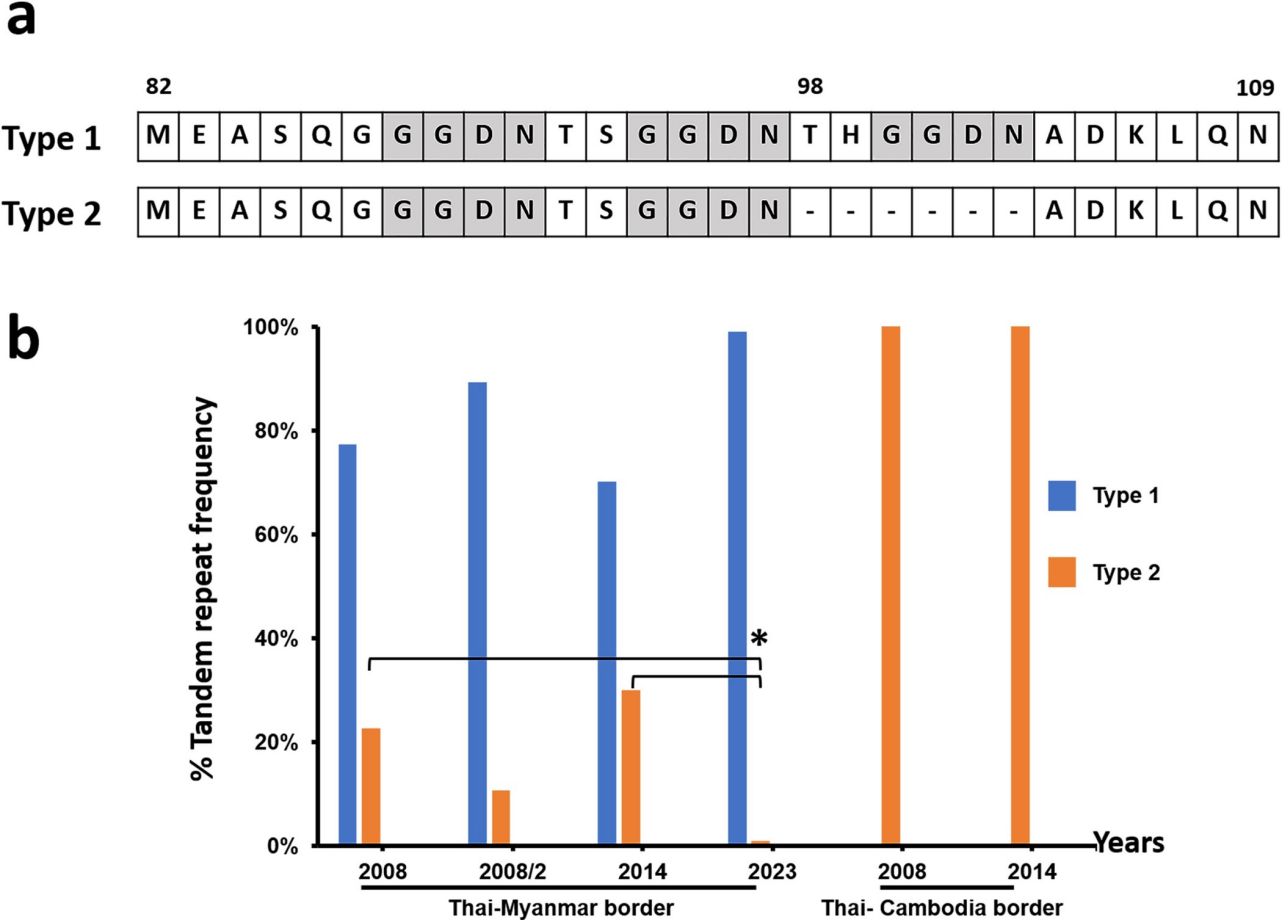
Prevalence of tandem repeat variants *Pvdhfr* in *P*. *vivax* isolates from a Kanchanaburi province, western, Thailand. Data were combined from previously published surveys on the Thai–Myanmar border and Thai–Cambodia borders between 2008 [20], 2008/2 [19], 2014 [20] and 2023 in this study. (a) Sequences alignment of tandem repeat variants in the *Pvdhfr* gene between amino acid positions 82 and 109. Type 1 contains three copies of GGDN repeats. Type 2 represents deletion of six amino acids from positions 98 to 103. GGDN repeats are shown in text highlight color. The amino acid deletions are shown as Dashes (-). (b) Prevalence of tandem repeat variants are shown as percentages on the y axis, while the x axis indicate time (in years). The difference in the tandem repeat variants frequencies between year was calculated by Chi square and 2-tailed Fisher’s exact tests with statistically significant difference at *P*-value < 0.001.

The tandem repeat region between the amino acid positions 82 and 109 was identified in the *Pvdhfr* gene (Fig 4A). The type 1, with three copies of GGDN repeats, is the majority of the isolates (99%), commonly occurring with quadruple-mutant *Pvdhfr* alleles [17, 19, 20]. Meanwhile, type 2, with a deletion of six amino acids, was detected in one isolate, and it usually co-existed with the double mutations (**R**_**58**_**N**_**117**_) [17, 19, 20]. Combined with previous surveys of the prevalence of tandem repeat region in *Pvdhfr* mutations from the Thai–Myanmar border, our results are consistent with previously reported studies [19, 20], where type 1 is predominant (Fig 4B, S4 Table). In contrast, type 2 was found in all isolates (100%) at the Thai–Cambodia border. At the Thai–Myanmar border, Type 2 has decreased (22.6% in 2008, 29.9% in 2014 and 1% in 2023) with statistical significance (Fig 4B, S4 Table). Tandem repeat variations in *Pvdhfr* are possibly linked with increased resistance to pyrimethamine resistance [19, 65]; however, the mechanism of these repeat regions that confer the risk of drug resistance remains unclear [20].

For *Pvdhps*, mutations at codon S382A, A383G, K512M/E, A553G, and V585A have been associated with resistance to sulfadoxine [16, 24, 34, 40]. Among these codons, the single (**G**_**383**_) and double (**G**_**383**_**G**_**553**_) mutant *Pvdhps* alleles are directly related to sulfadoxine resistance by which a disruption in the affinity between sulfadoxine-binding site in *P*. *vivax* [34]. G383 and G553 codons prevailed in 95% of the isolates, similar to that reported by other studies that these codon mutations were the majority from the Thai–Myanmar border [20, 21]. In contrast, codon **G**_**383**_ was more prevalent from the Thai–Cambodia border (Fig 3E). The prevalence of **G**_**383**_ at the Thai–Myanmar border between 2011, 2014 and 2023 increased from 46.3%, 46.3% to 95%, respectively with statistical significance (Fig 3E, S2 Table). Haplotype analysis of *Pvdhps* revealed a high prevalence of double mutant (**G**_**383**_**G**_**553**_) in this study (48%), a result congruent with previous studies on the Thai–Myanmar border [19, 20]. In contrast, only WT and single mutant (**G**_**383**_) were detected (Fig 3F). In this study, high prevalence of the triple mutant (**G**_**383**_**M**_**512**_**G**_**553**_) *Pvdhps* alleles were observed in 47%, which is inconsistent with those previous reports at the same border region [19, 20]. Double mutant (**G**_**383**_**G**_**553**_) has been linked with high-level resistance in sulfadoxine compared to the wildtype [69]. Though **M**_**512**_ allele might not involve with sulfadoxine resistance, the double (**G**_**383**_**G**_**553**_) and triple (**G**_**383**_**M**_**512**_**G**_**553**_) *Pvdhps* mutant alleles that carried mutation at codon 383 and 553 were highly prevalent in this region. It was indicated that the resistance of the *P*. *vivax* parasite to sulfadoxine at the Thai–Myanmar border is still high.

Distributions of *Pvdhfr* and *Pvdhps* allelic combinations among *P*. *vivax* isolates were identified in this study. The combination of *Pvdhfr* quadruple mutant (**I**_**57**_**R**_**58**_**M**_**61**_**T**_**117**_) and double-mutant (**G**_**383**_**G**_**553**_) *Pvdhps* alleles–forming sextuple mutated haplotypes was detected as the most prevalence of 48% (Fig 1). The result was concordant with previous reports, in which sextuple mutated haplotypes in the isolates were found in most samples obtained from areas along the Thai–Myanmar border [19]. The clinical response to SP depends on the *Pvdhfr* and *Pvdhps* genotype [65]. Sextuple mutations have been associated with high-grade drug resistance, which contributed to the clinical failure of SP treatment in *P*. *vivax* infection [70]. Vivax parasites harboring the sextuple mutated haplotypes were eliminated more slowly after receiving SP treatment than less-mutated parasites. Moreover, early treatment failures in patients were linked to infection with multiple mutants in the combination of the *Pvdhfr* and *Pvdhps* genes [70]. The mutant *Pvdhps* **M**_**512**_ allele was found in a high frequency (47%) in this study, while it was not found in the previous study [19]. Therefore, a triple-mutant allele (**G**_**383**_**M**_**512**_**G**_**553**_) of *Pvdhps* combined a quadruple-mutant (a) (**I**_**57**_**R**_**58**_**M**_**61**_**T**_**117**_), a quadruple-mutant (b) (**L**_**57**_**R**_**58**_**M**_**61**_**T**_**117**_), and a double mutant allele (**R**_**58**_**N**_**117**_) of *Pvdhfr* was detected in 35%, 11% and 1%, respectively. In contrast, a double mutant (**G**_**383**_**G**_**553**_) *Pvdhps* combined with a double mutant (**R**_**58**_**N**_**117**_) or a quadruple mutant (b) (**L**_**57**_**R**_**58**_**M**_**61**_**T**_**117**_) *Pvdhfr* alleles were found at 10.7% and 7.1%, respectively from the previous survey [19]. Additionally, the LD analysis showed statistically significant intragenic and intergenic associations among the SNP pairs between the *Pvdhfr* and *Pvdhps* genes (Fig 2). These results indicate that the alleles located in both genes were dependent on each other. Even though SP has never been prescribed against *P*. *vivax* in Thailand, the high prevalence of sextuple and septuple mutated haplotypes the *Pvdhfr* and *Pvdhps* genes was observed in *P*. *vivax* isolates during this study. Sextuple mutations found in this study indicated that the selection pressure came from widespread use of SP in this region. A combination of SP with other antimalarial drugs has been recommended to treat uncomplicated falciparum malaria, where co-infection with *P*. *falciparum* and *P*. *vivax* is common. The use of SP may have placed the *P*. *vivax* parasite under SP drug selection pressure [70, 71]. SP mutations reflect that *P*. *vivax* has had exposure to SP in the past. Moreover, it may be a consequence of misdiagnosis of malaria in the absence of clinical suspicion. Misdiagnosed patients might receive unnecessary medication. Self-treatment of *P*. *vivax* infection with SP might be associated with SP resistance in endemic areas [20]. Labor migration of the population from neighboring countries along the international borders might also cause the introduction of resistant parasites. This study’s results, combined with a previously published survey on the Thai–Myanmar border, demonstrated that diverse patterns of *Pvdhfr* and *Pvdhps* mutant alleles in *P*. *vivax* were found and changed over time in this malaria-endemic region. The prevalence and patterns of combined mutations in *Pvdhfr* and *Pvdhps* in this study indicated that SP resistance in *P*. *vivax* is still prevalent in these areas. The surveillance of the distribution of combined *Pvdhfr* and *Pvdhps* polymorphisms is thus essential to evaluate the development and spread of SP resistance in *P*. *vivax* in this region.

Though the prevalence of drug resistance in *P*. *vivax* parasites on the Thai-Myanmar border in this study was congruent with previously published results from 2008 to 2014 in Thailand, the limitations of this study should be considered. The study samples were included from one geographical area. Only symptomatic patients who had presented to an accessible hospital were obtained for the analysis. The samples from the different provinces within border regions should be enrolled to represent the true prevalence of resistance alleles. The data reported in this study may not be construed as indicating the overall prevalence of malaria in Thailand. Thus, ongoing and comprehensive molecular surveillance, together with using whole-genome sequencing for further studies targeting multiple markers, is required to inform policymakers and enable the stated Thai malaria elimination strategy.

## Conclusions

This study reported an up-to-date overview of the prevalence of three potential drug-resistant markers, *Pvmdr1*, *Pvdhfr*, and *Pvdhps* in *P*. *vivax* isolates from a Kanchanaburi province, along the Thai–Myanmar border. High mutations in these drug-resistance genes are prevalent in this region. Further assessment of drug resistance from different geographical locations and the expansion of the sample size would be necessary to provide a more representative conclusion of the frequencies of the mutations. Thus, these data will serve as a baseline for further monitoring of drug-resistant malaria.

## Supporting information

S1 Table Analysis of SNPs in *P*. *vivax* genetic markers. (XLSX)

S2 Table Prevalence of target mutation conferring antimalarial drug resistance in *P*. *vivax* isolates from a Kanchanaburi province, Thailand collected between January to May 2023, combined from previously published surveys on the Thai–Myanmar and Thai–Cambodia borders between 2008, 2010, 2011 and 2014. (PDF)

S3 Table Prevalence of *P*. *vivax Pvmdr1*, *Pvdhfr* and *Pvdhps* haplotypes in isolates collected from a Kanchanaburi province, Thailand during January to May 2023, combined from previously published surveys on the Thai–Myanmar and Thai–Cambodia borders between 2008, 2008/2 and 2014. (PDF)

S4 Table Prevalence of tandem repeat variants *Pvdhfr* in isolates collected from a Kanchanaburi province, Thailand during January to May 2023, combined from previously published surveys on the Thai–Myanmar and Thai–Cambodia borders between 2008, 2008/2 and 2014. (PDF)

S1 File The gene sequences reported in this study were shown in FASTA format. (RAR)

## References

[1] World Health Organization. World malaria report 2022, Avaliable from: https://www.who.int/teams/global-malaria-programme/reports/world-malaria-report-2022, [accessed 24 November 2023].

[2] Mala W, Wilairatana P, Samerjai C, Masangkay FR, Kotepui KU, Kotepui M. Prevalence of Signs of Severity Identified in the Thai Population with Malaria: A Systematic Review and Meta-Analysis. Int J Environ Res Public Health. 2022;19(3). Epub 20220121. doi: 10.3390/ijerph19031196 ; PubMed Central PMCID: PMC8834971. 35162229 PMC8834971

[3] Chang HH, Chang MC, Kiang M, Mahmud AS, Ekapirat N, Engø-Monsen K, et al. Low parasite connectivity among three malaria hotspots in Thailand. Sci Rep. 2021;11(1):23348. Epub 20211202. doi: 10.1038/s41598-021-02746-6 ; PubMed Central PMCID: PMC8640040. 34857842 PMC8640040

[4] Bureau of Vector-Borne Diseases. Annual Report 2020. Nonthaburi: Department of Disease Control, Ministry of Public Health. 2020.

[5] Zeyrek FY, Kurcer MA, Zeyrek D, Simsek Z. Parasite density and serum cytokine levels in *Plasmodium vivax* malaria in Turkey. Parasite Immunol. 2006;28(5):201–7. doi: 10.1111/j.1365-3024.2006.00822.x . 16629705

[6] Price RN, Tjitra E, Guerra CA, Yeung S, White NJ, Anstey NM. Vivax malaria: neglected and not benign. Am J Trop Med Hyg. 2007;77(6 Suppl):79–87. ; PubMed Central PMCID: PMC2653940. 18165478 PMC2653940

[7] Bureau of Vector-Borne Disease. National Strategic Plan for Malaria Control and Elimination in Thailand 2017–2026. Nonthaburi: Ministry of Public Health; 2017.

[8] Bhumiratana A, Intarapuk A, Sorosjinda-Nunthawarasilp P, Maneekan P, Koyadun S. Border malaria associated with multidrug resistance on Thailand-Myanmar and Thailand-Cambodia borders: transmission dynamic, vulnerability, and surveillance. Biomed Res Int. 2013;2013:363417. Epub 20130625. doi: 10.1155/2013/363417 ; PubMed Central PMCID: PMC3707221. 23865048 PMC3707221

[9] Pukrittayakamee S, Tarning J, Jittamala P, Charunwatthana P, Lawpoolsri S, Lee SJ, et al. Pharmacokinetic interactions between primaquine and chloroquine. Antimicrob Agents Chemother. 2014;58(6):3354–9. Epub 20140331. doi: 10.1128/AAC.02794-13 ; PubMed Central PMCID: PMC4068454. 24687509 PMC4068454

[10] Bureau of Vector-Borne Disease. Guidelines for the Treatment of Malaria in Thailand 2021. Nonthaburi: Ministry of Public Health; 2021.

[11] Price RN, von Seidlein L, Valecha N, Nosten F, Baird JK, White NJ. Global extent of chloroquine-resistant *Plasmodium vivax*: a systematic review and meta-analysis. Lancet Infect Dis. 2014;14(10):982–91. Epub 20140908. doi: 10.1016/s1473-3099(14)70855-2 ; PubMed Central PMCID: PMC4178238. 25213732 PMC4178238

[12] Vijaykadga S, Rojanawatsirivej C, Congpoung K, Wilairatana P, Satimai W, Uaekowitchai C, et al. Assessment of therapeutic efficacy of chloroquine for vivax malaria in Thailand. Southeast Asian journal of tropical medicine and public health. 2004;35:566–9. 15689067

[13] Rijken MJ, Boel ME, Russell B, Imwong M, Leimanis ML, Phyo AP, et al. Chloroquine resistant vivax malaria in a pregnant woman on the western border of Thailand. Malar J. 2011;10:113. Epub 20110505. doi: 10.1186/1475-2875-10-113 ; PubMed Central PMCID: PMC3112451. 21545737 PMC3112451

[14] World Health Organization. Strategic framework for malaria prevention and control during pregnancy in the Africa Region, Regional Office for Africa, Brazzaville, AFR/MAL/04/01. 2004.

[15] Müller IB, Hyde JE. Folate metabolism in human malaria parasites—75 years on. Mol Biochem Parasitol. 2013;188(1):63–77. Epub 20130315. doi: 10.1016/j.molbiopara.2013.02.008 . 23500968

[16] Imwong M, Pukrittayakamee S, Rénia L, Letourneur F, Charlieu JP, Leartsakulpanich U, et al. Novel point mutations in the dihydrofolate reductase gene of *Plasmodium vivax*: evidence for sequential selection by drug pressure. Antimicrob Agents Chemother. 2003;47(5):1514–21. doi: 10.1128/aac.47.5.1514–1521.2003 ; PubMed Central PMCID: PMC153323. 12709316 PMC153323

[17] Alam MT, Bora H, Bharti PK, Saifi MA, Das MK, Dev V, et al. Similar trends of pyrimethamine resistance-associated mutations in *Plasmodium vivax* and *P*. *falciparum*. Antimicrob Agents Chemother. 2007;51(3):857–63. Epub 20061228. doi: 10.1128/aac.01200-06 ; PubMed Central PMCID: PMC1803105. 17194833 PMC1803105

[18] Pukrittayakamee S, Chantra A, Simpson JA, Vanijanonta S, Clemens R, Looareesuwan S, et al. Therapeutic responses to different antimalarial drugs in vivax malaria. Antimicrob Agents Chemother. 2000;44(6):1680–5. doi: 10.1128/AAC.44.6.1680-1685.2000 ; PubMed Central PMCID: PMC89932. 10817728 PMC89932

[19] Lu F, Lim CS, Nam DH, Kim K, Lin K, Kim TS, et al. Mutations in the antifolate-resistance-associated genes dihydrofolate reductase and dihydropteroate synthase in *Plasmodium vivax* isolates from malaria-endemic countries. Am J Trop Med Hyg. 2010;83(3):474–9. doi: 10.4269/ajtmh.2010.10–0004 ; PubMed Central PMCID: PMC2929037. 20810806 PMC2929037

[20] Tantiamornkul K, Pumpaibool T, Piriyapongsa J, Culleton R, Lek-Uthai U. The prevalence of molecular markers of drug resistance in *Plasmodium vivax* from the border regions of Thailand in 2008 and 2014. Int J Parasitol Drugs Drug Resist. 2018;8(2):229–37. Epub 20180412. doi: 10.1016/j.ijpddr.2018.04.003 ; PubMed Central PMCID: PMC6039358. 29677637 PMC6039358

[21] Thongdee P, Kuesap J, Rungsihirunrat K, Tippawangkosol P, Mungthin M, Na-Bangchang K. Distribution of dihydrofolate reductase (*dhfr*) and dihydropteroate synthase (*dhps*) mutant alleles in *Plasmodium vivax* isolates from Thailand. Acta Trop. 2013;128(1):137–43. Epub 20130721. doi: 10.1016/j.actatropica.2013.07.005 . 23880285

[22] Buyon LE, Elsworth B, Duraisingh MT. The molecular basis of antimalarial drug resistance in *Plasmodium vivax*. Int J Parasitol Drugs Drug Resist. 2021;16:23–37. Epub 20210426. doi: 10.1016/j.ijpddr.2021.04.002 ; PubMed Central PMCID: PMC8113647. 33957488 PMC8113647

[23] Suwanarusk R, Russell B, Chavchich M, Chalfein F, Kenangalem E, Kosaisavee V, et al. Chloroquine resistant *Plasmodium vivax*: in vitro characterisation and association with molecular polymorphisms. PLoS One. 2007;2(10):e1089. Epub 20071031. doi: 10.1371/journal.pone.0001089 ; PubMed Central PMCID: PMC2034531. 17971853 PMC2034531

[24] Mint Lekweiry K, Ould Mohamed Salem Boukhary A, Gaillard T, Wurtz N, Bogreau H, Hafid JE, et al. Molecular surveillance of drug-resistant *Plasmodium vivax* using *pvdhfr*, *pvdhps* and *pvmdr1* markers in Nouakchott, Mauritania. J Antimicrob Chemother. 2012;67(2):367–74. Epub 20111114. doi: 10.1093/jac/dkr464 . 22086859

[25] Ferreira MU, Nobrega de Sousa T, Rangel GW, Johansen IC, Corder RM, Ladeia-Andrade S, et al. Monitoring *Plasmodium vivax* resistance to antimalarials: Persisting challenges and future directions. Int J Parasitol Drugs Drug Resist. 2021;15:9–24. Epub 20201205. doi: 10.1016/j.ijpddr.2020.12.001 ; PubMed Central PMCID: PMC7770540. 33360105 PMC7770540

[26] Price RN, Auburn S, Marfurt J, Cheng Q. Phenotypic and genotypic characterisation of drug-resistant *Plasmodium vivax*. Trends Parasitol. 2012;28(11):522–9. Epub 20121005. doi: 10.1016/j.pt.2012.08.005 ; PubMed Central PMCID: PMC4627502. 23044287 PMC4627502

[27] Marfurt J, de Monbrison F, Brega S, Barbollat L, Müller I, Sie A, et al. Molecular markers of *in vivo Plasmodium vivax* resistance to amodiaquine plus sulfadoxine-pyrimethamine: mutations in *pvdhfr* and *pvmdr1*. J Infect Dis. 2008;198(3):409–17. doi: 10.1086/589882 . 18582193

[28] Anantabotla VM, Antony HA, Parija SC, Rajkumari N, Kini JR, Manipura R, et al. Polymorphisms in genes associated with drug resistance of *Plasmodium vivax* in India. Parasitol Int. 2019;70:92–7. Epub 20190302. doi: 10.1016/j.parint.2019.03.001 . 30836136

[29] Rungsihirunrat K, Muhamad P, Chaijaroenkul W, Kuesap J, Na-Bangchang K. *Plasmodium vivax* drug resistance genes; *Pvmdr1* and *Pvcrt-o* polymorphisms in relation to chloroquine sensitivity from a malaria endemic area of Thailand. Korean J Parasitol. 2015;53(1):43–9. Epub 20150227. doi: 10.3347/kjp.2015.53.1.43 ; PubMed Central PMCID: PMC4384798. 25748708 PMC4384798

[30] Imwong M, Pukrittayakamee S, Pongtavornpinyo W, Nakeesathit S, Nair S, Newton P, et al. Gene amplification of the multidrug resistance 1 gene of *Plasmodium vivax* isolates from Thailand, Laos, and Myanmar. Antimicrob Agents Chemother. 2008;52(7):2657–9. Epub 20080428. doi: 10.1128/aac.01459-07 ; PubMed Central PMCID: PMC2443893. 18443118 PMC2443893

[31] Petersen I, Eastman R, Lanzer M. Drug-resistant malaria: molecular mechanisms and implications for public health. FEBS Lett. 2011;585(11):1551–62. Epub 20110423. doi: 10.1016/j.febslet.2011.04.042 . 21530510

[32] Gregson A, Plowe CV. Mechanisms of resistance of malaria parasites to antifolates. Pharmacol Rev. 2005;57(1):117–45. doi: 10.1124/pr.57.1.4 . 15734729

[33] Tjitra E, Baker J, Suprianto S, Cheng Q, Anstey NM. Therapeutic efficacies of artesunate-sulfadoxine-pyrimethamine and chloroquine-sulfadoxine-pyrimethamine in vivax malaria pilot studies: relationship to *Plasmodium vivax dhfr* mutations. Antimicrob Agents Chemother. 2002;46(12):3947–53. doi: 10.1128/aac.46.12.3947–3953.2002 ; PubMed Central PMCID: PMC132782. 12435700 PMC132782

[34] Korsinczky M, Fischer K, Chen N, Baker J, Rieckmann K, Cheng Q. Sulfadoxine resistance in *Plasmodium vivax* is associated with a specific amino acid in dihydropteroate synthase at the putative sulfadoxine-binding site. Antimicrob Agents Chemother. 2004;48(6):2214–22. doi: 10.1128/aac.48.6.2214–2222.2004 ; PubMed Central PMCID: PMC415609. 15155224 PMC415609

[35] de Pécoulas PE, Tahar R, Yi P, Thai KH, Basco LK. Genetic variation of the dihydrofolate reductase gene in *Plasmodium vivax* in Snoul, northeastern Cambodia. Acta Trop. 2004;92(1):1–6. doi: 10.1016/j.actatropica.2004.03.011 . 15301969

[36] Asih PB, Marantina SS, Nababan R, Lobo NF, Rozi IE, Sumarto W, et al. Distribution of *Plasmodium vivax pvdhfr* and *pvdhps* alleles and their association with sulfadoxine-pyrimethamine treatment outcomes in Indonesia. Malar J. 2015;14:365. Epub 20150922. doi: 10.1186/s12936-015-0903-0 ; PubMed Central PMCID: PMC4580362. 26395428 PMC4580362

[37] Joy S, Ghosh SK, Achur RN, Gowda DC, Surolia N. Presence of novel triple mutations in the *pvdhfr* from *Plasmodium vivax* in Mangaluru city area in the southwestern coastal region of India. Malar J. 2018;17(1):167. Epub 20180416. doi: 10.1186/s12936-018-2316-3 ; PubMed Central PMCID: PMC5902849. 29661235 PMC5902849

[38] Barnadas C, Tichit M, Bouchier C, Ratsimbasoa A, Randrianasolo L, Raherinjafy R, et al. Plasmodium *vivax dhfr* and *dhps* mutations in isolates from Madagascar and therapeutic response to sulphadoxine-pyrimethamine. Malar J. 2008;7:35. Epub 20080226. doi: 10.1186/1475-2875-7-35 ; PubMed Central PMCID: PMC2268703. 18302746 PMC2268703

[39] Barnadas C, Timinao L, Javati S, Iga J, Malau E, Koepfli C, et al. Significant geographical differences in prevalence of mutations associated with *Plasmodium falciparum* and *Plasmodium vivax* drug resistance in two regions from Papua New Guinea. Malar J. 2015;14:399. Epub 20151009. doi: 10.1186/s12936-015-0879-9 ; PubMed Central PMCID: PMC4600278. 26452541 PMC4600278

[40] Hawkins VN, Joshi H, Rungsihirunrat K, Na-Bangchang K, Sibley CH. Antifolates can have a role in the treatment of *Plasmodium vivax*. Trends Parasitol. 2007;23(5):213–22. Epub 20070326. doi: 10.1016/j.pt.2007.03.002 . 17368986

[41] Kitvatanachai S, Rhongbutsri P. Malaria in asymptomatic migrant workers and symptomatic patients in Thamaka District, Kanchanaburi Province, Thailand. Asian Pacific Journal of Tropical Disease. 2012;2:S374–S7.

[42] Department of Disease Control. Thailand malaria elimination program; Bangkok, Thailand. 2023.

[43] Nguitragool W, Mueller I, Kumpitak C, Saeseu T, Bantuchai S, Yorsaeng R, et al. Very high carriage of gametocytes in asymptomatic low-density *Plasmodium falciparum* and *P*. *vivax* infections in western Thailand. Parasit Vectors. 2017;10(1):512. Epub 20171024. doi: 10.1186/s13071-017-2407-y ; PubMed Central PMCID: PMC5655986. 29065910 PMC5655986

[44] World Health Organization. Malaria parasite counting. No. WHO/HTM/GMP/MM/SOP/2016.09. 2016.

[45] Singh B, Bobogare A, Cox-Singh J, Snounou G, Abdullah MS, Rahman HA. A genus- and species-specific nested polymerase chain reaction malaria detection assay for epidemiologic studies. Am J Trop Med Hyg. 1999;60(4):687–92. doi: 10.4269/ajtmh.1999.60.687 . 10348249

[46] Snounou G, Viriyakosol S, Zhu XP, Jarra W, Pinheiro L, do Rosario VE, et al. High sensitivity of detection of human malaria parasites by the use of nested polymerase chain reaction. Mol Biochem Parasitol. 1993;61(2):315–20. doi: 10.1016/0166-6851(93)90077-b . 8264734

[47] Singh B, Kim Sung L, Matusop A, Radhakrishnan A, Shamsul SS, Cox-Singh J, et al. A large focus of naturally acquired *Plasmodium knowlesi* infections in human beings. Lancet. 2004;363(9414):1017–24. doi: 10.1016/s0140-6736(04)15836-4 . 15051281

[48] Barnadas C, Kent D, Timinao L, Iga J, Gray LR, Siba P, et al. A new high-throughput method for simultaneous detection of drug resistance associated mutations in *Plasmodium vivax dhfr*, *dhps* and *mdr1* genes. Malar J. 2011;10:282. Epub 20110924. doi: 10.1186/1475-2875-10-282 ; PubMed Central PMCID: PMC3192712. 21943242 PMC3192712

[49] Lu F, Lim CS, Nam DH, Kim K, Lin K, Kim TS, et al. Genetic polymorphism in *pvmdr1* and *pvcrt-o* genes in relation to *in vitro* drug susceptibility of *Plasmodium vivax* isolates from malaria-endemic countries. Acta Trop. 2011;117(2):69–75. Epub 20101008. doi: 10.1016/j.actatropica.2010.08.011 . 20933490

[50] Ding S, Ye R, Zhang D, Sun X, Zhou H, McCutchan TF, et al. Anti-folate combination therapies and their effect on the development of drug resistance in *Plasmodium vivax*. Sci Rep. 2013;3:1008. Epub 20130107. doi: 10.1038/srep01008 ; PubMed Central PMCID: PMC3538286. 23301149 PMC3538286

[51] Barrett JC, Fry B, Maller J, Daly MJ. Haploview: analysis and visualization of LD and haplotype maps. Bioinformatics. 2005;21(2):263–5. Epub 20040805. doi: 10.1093/bioinformatics/bth457 . 15297300

[52] Baird JK, Basri H, Purnomo, Bangs MJ, Subianto B, Patchen LC, et al. Resistance to chloroquine by *Plasmodium vivax* in Irian Jaya, Indonesia. Am J Trop Med Hyg. 1991;44(5):547–52. doi: 10.4269/ajtmh.1991.44.547 . 1676566

[53] Fryauff DJ, Tuti S, Mardi A, Masbar S, Patipelohi R, Leksana B, et al. Chloroquine-resistant *Plasmodium vivax* in transmigration settlements of West Kalimantan, Indonesia. Am J Trop Med Hyg. 1998;59(4):513–8. doi: 10.4269/ajtmh.1998.59.513 . 9790420

[54] Phillips EJ, Keystone JS, Kain KC. Failure of combined chloroquine and high-dose primaquine therapy for *Plasmodium vivax* malaria acquired in Guyana, South America. Clin Infect Dis. 1996;23(5):1171–3. doi: 10.1093/clinids/23.5.1171 . 8922821

[55] Baird JK. Resistance to therapies for infection by *Plasmodium vivax*. Clin Microbiol Rev. 2009;22(3):508–34. doi: 10.1128/cmr.00008-09 ; PubMed Central PMCID: PMC2708388. 19597012 PMC2708388

[56] Krudsood S, Tangpukdee N, Muangnoicharoen S, Thanachartwet V, Luplertlop N, Srivilairit S, et al. Clinical efficacy of chloroquine versus artemether-lumefantrine for *Plasmodium vivax* treatment in Thailand. Korean J Parasitol. 2007;45(2):111–4. doi: 10.3347/kjp.2007.45.2.111 ; PubMed Central PMCID: PMC2526312. 17570973 PMC2526312

[57] Fidock DA, Nomura T, Talley AK, Cooper RA, Dzekunov SM, Ferdig MT, et al. Mutations in the *P*. *falciparum* digestive vacuole transmembrane protein *PfCRT* and evidence for their role in chloroquine resistance. Mol Cell. 2000;6(4):861–71. doi: 10.1016/s1097-2765(05)00077-8 ; PubMed Central PMCID: PMC2944663. 11090624 PMC2944663

[58] Suwanarusk R, Chavchich M, Russell B, Jaidee A, Chalfein F, Barends M, et al. Amplification of *pvmdr1* associated with multidrug-resistant *Plasmodium vivax*. J Infect Dis. 2008;198(10):1558–64. doi: 10.1086/592451 ; PubMed Central PMCID: PMC4337975. 18808339 PMC4337975

[59] Shalini S, Chaudhuri S, Sutton PL, Mishra N, Srivastava N, David JK, et al. Chloroquine efficacy studies confirm drug susceptibility of *Plasmodium vivax* in Chennai, India. Malar J. 2014;13:129. Epub 20140331. doi: 10.1186/1475-2875-13-129 ; PubMed Central PMCID: PMC4021252. 24685286 PMC4021252

[60] Brega S, Meslin B, de Monbrison F, Severini C, Gradoni L, Udomsangpetch R, et al. Identification of the *Plasmodium vivax* mdr-like gene (*pvmdr1*) and analysis of single-nucleotide polymorphisms among isolates from different areas of endemicity. J Infect Dis. 2005;191(2):272–7. Epub 20041209. doi: 10.1086/426830 . 15609238

[61] Gomes LR, Almeida-de-Oliveira NK, de Lavigne AR, de Lima SR, de Pina-Costa A, Brasil P, et al. *Plasmodium vivax mdr1* genotypes in isolates from successfully cured patients living in endemic and non-endemic Brazilian areas. Malar J. 2016;15:96. Epub 20160218. doi: 10.1186/s12936-016-1141-9 ; PubMed Central PMCID: PMC4758108. 26887935 PMC4758108

[62] Barnadas C, Ratsimbasoa A, Tichit M, Bouchier C, Jahevitra M, Picot S, et al. *Plasmodium vivax* resistance to chloroquine in Madagascar: clinical efficacy and polymorphisms in *pvmdr1* and *pvcrt-o* genes. Antimicrob Agents Chemother. 2008;52(12):4233–40. Epub 20080922. doi: 10.1128/aac.00578-08 ; PubMed Central PMCID: PMC2592859. 18809933 PMC2592859

[63] Roesch C, Mairet-Khedim M, Kim S, Lek D, Popovici J, Witkowski B. Impact of the first-line treatment shift from dihydroartemisinin/piperaquine to artesunate/mefloquine on *Plasmodium vivax* drug susceptibility in Cambodia. J Antimicrob Chemother. 2020;75(7):1766–71. doi: 10.1093/jac/dkaa092 ; PubMed Central PMCID: PMC7303819. 32211790 PMC7303819

[64] Wongsrichanalai C, Prajakwong S, Meshnick SR, Shanks GD, Thimasarn K. Mefloquine—its 20 years in the Thai Malaria Control Program. Southeast Asian J Trop Med Public Health. 2004;35(2):300–8. . 15691128

[65] Rungsihirunrat K, Na-Bangchang K, Hawkins VN, Mungthin M, Sibley CH. Sensitivity to antifolates and genetic analysis of *Plasmodium vivax* isolates from Thailand. Am J Trop Med Hyg. 2007;76(6):1057–65. . 17556611

[66] Auliff AM, Adams JH, O’Neil MT, Cheng Q. Defining the role of mutations in *Plasmodium vivax* dihydrofolate reductase-thymidylate synthase gene using an episomal *Plasmodium falciparum* transfection system. Antimicrob Agents Chemother. 2010;54(9):3927–32. Epub 20100621. doi: 10.1128/aac.00628-10 ; PubMed Central PMCID: PMC2934960. 20566761 PMC2934960

[67] Hastings MD, Maguire JD, Bangs MJ, Zimmerman PA, Reeder JC, Baird JK, et al. Novel *Plasmodium vivax dhfr* alleles from the Indonesian Archipelago and Papua New Guinea: association with pyrimethamine resistance determined by a *Saccharomyces cerevisiae* expression system. Antimicrob Agents Chemother. 2005;49(2):733–40. doi: 10.1128/aac.49.2.733–740.2005 ; PubMed Central PMCID: PMC547327. 15673758 PMC547327

[68] Zhao Y, Wang L, Soe MT, Aung PL, Wei H, Liu Z, et al. Molecular surveillance for drug resistance markers in *Plasmodium vivax* isolates from symptomatic and asymptomatic infections at the China-Myanmar border. Malar J. 2020;19(1):281. Epub 20200805. doi: 10.1186/s12936-020-03354-x ; PubMed Central PMCID: PMC7409419. 32758218 PMC7409419

[69] Yogavel M, Nettleship JE, Sharma A, Harlos K, Jamwal A, Chaturvedi R, et al. Structure of 6-hydroxymethyl-7,8-dihydropterin pyrophosphokinase-dihydropteroate synthase from *Plasmodium vivax* sheds light on drug resistance. J Biol Chem. 2018;293(39):14962–72. Epub 20180813. doi: 10.1074/jbc.RA118.004558 ; PubMed Central PMCID: PMC6166723. 30104413 PMC6166723

[70] Imwong M, Pukrittayakamee S, Cheng Q, Moore C, Looareesuwan S, Snounou G, et al. Limited polymorphism in the dihydropteroate synthetase gene (*dhps*) of *Plasmodium vivax* isolates from Thailand. Antimicrob Agents Chemother. 2005;49(10):4393–5. doi: 10.1128/aac.49.10.4393–4395.2005 ; PubMed Central PMCID: PMC1251524. 16189131 PMC1251524

[71] Thimasarn K. Malaria Control Program in Thailand. Bangkok, Thailand: Malaria Division, Department of Communicable Disease Control, Ministry of Public Health, Thailand. 1999;88:102.

