# Supplementary material for: Drug resistance markers in Plasmodium vivax isolates from a Kanchanaburi province, Thailand between January to May 2023
Source: PLoS One. 2024 Jul 5;19(7):e0304337. doi: 10.1371/journal.pone.0304337 (PMC11226124; doi:10.1371/journal.pone.0304337)
Supplement: S2 Table — (PDF) [file pone.0304337.s002.pdf]

S2 Table. Prevalence of target mutation conferring antimalarial drug resistance in *P. vivax* isolates from a Kanchanaburi province, Thailand collected between January to May 2023, combined from previously published surveys on the Thai–Myanmar and Thai–Cambodia borders between 2008, 2010, 2011 and 2014.

**Gene**	**Codon mutation**	**Number of isolates (%)**					***P*-value**	**Number of isolates (%)**		
		**Thai-Myanmar**						**Thai-Cambodia**		
		**2008**	**2010**	**2011**	**2014**	**2023**		**2008**	**2011**	**2014**
Pvmdr1		*n = 82*	*n = 30*		*n = 73*	*n = 100*		*n = 44*		*n = 14*
	958	82 (100)	30 (100)	NA	73 (100)	100 (100)	a*: NR, NR, NR	44 (100)	NA	14 (100)
	976	26 (31.7)	7 (23.3)	NA	8 (11)	21 (21)	a*: 0.126, 0.803, 0.100	42 (95.5)	NA	9 (64.3)
	1076	51 (62.2)	16 (53.3)	NA	29 (39.7)	23 (23)	a*: <0.001, 0.003, 0.020	40 (90.9)	NA	14 (100)
Pvdhfr		*n = 84*			*n = 77*	*n = 100*		*n = 60*	*n = 65*	*n = 17*
	57	65 (77.4)	NA	56 (93.3), *n = 60*	54 (70.1)	95 (95)	b*: <0.001, 0.729, <0.001	0	3 (4.6)	0
	58	84 (100)	NA	66 (100), *n = 66*	77 (100)	96 (96)	b*: 0.127, 0.152, 0.133	60 (100)	65 (100)	17 (100)
	61	65 (77.4)	NA	17 (25.8), *n = 66*	54 (70.1)	95 (95)	b*: <0.001, <0.001, <0.001	0	37 (64.9), *n = 57*	0
	117	84 (100)	NA	63 (100), *n = 63*	77 (100)	96 (96)	b*: 0.127, 0.159, 0.133	60 (100)	55 (100), *n = 55*	17 (100)
Pvdhps		*n = 86*		*n = 67*	*n = 80*	*n = 100*		*n = 66*		*n = 8*
	382	13 (15.1)	NA	NA	24 (30)	0	c*: <0.001, <0.001	0	NA	0
	383	83 (96.5)	NA	31 (46.3)	37 (46.3)	95 (95)	c*: 0.727, <0.001, <0.001	31 (47)	17 (26.2), *n = 65*	8 (100)
	512	0	NA	NA	4 (5)	47 (47)	c*: <0.001, <0.001	0	NA	0
	553	72 (83.7)	NA	0	75 (93.8)	95 (95)	c*: 0.015, <0.001, 0.753	0	1 (2.1), *n= 47*	0

a*: *P* value 2008 *vs* 2023; 2010 *vs* 2023; 2014 *vs* 2023, respectively
b*: *P* value 2008 *vs* 2023; 2011 *vs* 2023; 2014 *vs* 2023, respectively
c*: *P* value 2008 *vs* 2023; 2011 *vs* 2023; 2014 *vs* 2023, respectively
All *P*-value were calculated by Chi square and 2-tailed Fisher's exact tests
Statistically significant difference between years at *P*-value < 0.001.
NA not available
NR not relevant to be calculated
