# Supplementary material for: Drug resistance markers in Plasmodium vivax isolates from a Kanchanaburi province, Thailand between January to May 2023
Source: PLoS One. 2024 Jul 5;19(7):e0304337. doi: 10.1371/journal.pone.0304337 (PMC11226124; doi:10.1371/journal.pone.0304337)
Supplement: S3 Table — (PDF) [file pone.0304337.s003.pdf]

S3 Table. Prevalence of *P. vivax Pvmdr1*, *Pvdhfr* and *Pvdhps* haplotypes in isolates collected from a Kanchanaburi province, Thailand during January to May 2023, combined from previously published surveys on the Thai–Myanmar and Thai–Cambodia borders between 2008, 2008/2 and 2014.

Gene	Haplotype	Codon	Number of isolates (%)				*P*-value	Number of isolates (%)	
			Thai-Myanmar					Thai-Cambodia	
			2008	2008/2	2014	2023		2008	2014
Pvmdr1		958/976/1076	*n = 82*		*n=73*	*n = 100*		*n=44*	*n=14*
	Wild-type	T/Y/F	0	NA	0	0	a*: NR, NR	0	0
	Single mutant	**M**/Y/F	29 (35.4)	NA	43 (58.9)	75 (75)	a*: <0.001, 0.032	0	0
	Double mutant (a)	**M**/**F**/F	2 (2.4)	NA	1 (1.4)	2 (2)	a*: 1.000, 1.000	4 (9.1)	0
	Double mutant (b)	**M**/Y/**L**	27 (32.9)	NA	22 (30.1)	4 (4)	a*: <0.001, <0.001	2 (4.5)	5 (35.7)
	Triple mutant	**M**/**F**/**L**	24 (29.3)	NA	7 (9.6)	19 (19)	a*: 0.117, 0.130	38 (86.4)	9 (64.3)
Pvdhfr		57/58/61/117	*n = 84*	*n = 28*	*n = 77*	*n = 100*		*n = 60*	*n =17*
	Wild-type	F/S/T/S	0	NA	0	4 (4)	b*: 0.127, 0.133	0	0
	Double mutant	F/**R**/T/**N**	19 (22.6)	3 (10.7)	23 (29.9)	1 (1)	b*: <0.001, 0.033, <0.001	60 (100)	17 (100)
	Quadruple mutant (a)	**I**/**R**/**M**/**T**	62 (73.8)	20 (71.4)	50 (64.9)	84 (84)	b*: 0.102, 0.170, 0.005	0	0
	Quadruple mutant (b)	**L**/**R**/**M**/**T**	3 (3.6)	4 (14.3)	4 (5.2)	11 (11)	b*: 0.092, 0.740, 0.188	0	0
Pvdhps		382/383/512/553	*n = 86*	*n = 28*	*n = 80*	*n = 100*		*n = 66*	*n = 8*
	Wild-type	S/A/K/A	2 (2.3)	1 (3.6)	0	5 (5)	b*: 0.454, 1.000, 0.067	35 (53)	0
	Single mutant	S/**G**/K/A	12 (14)	3 (10.7)	5 (6.3)	0	b*: <0.001, 0.010, 0.016	31 (47)	8 (100)
	Double mutant	S/**G**/K/**G**	59 (68.6)	19 (67.9)	49 (61.3)	48 (48)	b*: 0.005, 0.086, 0.098	0	0
	Triple mutant	S/**G**/**M**/**G**	0	1 (3.6)	1 (1.3)	47 (47)	b*: <0.001, <0.001, <0.001	0	0

Mutant amino acids are shown in bold with underlined letters
a*: *P* value 2008 *vs* 2023; 2014 *vs* 2023, respectively
b*: *P* value 2008 *vs* 2023; 2008/2 *vs* 2023; 2014 *vs* 2023, respectively
All *P*-value were calculated by Chi square and 2-tailed Fisher's exact tests
Statistically significant difference between years at *P*-value < 0.001.
NA not available
NR not relevant to be calculated
