# Supplementary material for: Drug resistance markers in Plasmodium vivax isolates from a Kanchanaburi province, Thailand between January to May 2023
Source: PLoS One. 2024 Jul 5;19(7):e0304337. doi: 10.1371/journal.pone.0304337 (PMC11226124; doi:10.1371/journal.pone.0304337)
Supplement: S4 Table — (PDF) [file pone.0304337.s004.pdf]

S4 Table. Prevalence of tandem repeat variants *Pvdhfr* in isolates collected from a Kanchanaburi province, Thailand during January to May 2023, combined from previously published surveys on the Thai–Myanmar and Thai–Cambodia borders between 2008, 2008/2 and 2014.

**Type**	**Number of isolates (%)**				***P*- value**	**Number of isolates (%)**	
	**Thai-Myanmar**					**Thai-Cambodia**	
	**2008**	**2008/2**	**2014**	**2023**		**2008**	**2014**
	n = 84	*n = 28*	*n = 77*	*n = 100*		*n = 60*	*n = 17*
1	65 (77.4)	25 (89.3)	54 (70.1)	99 (99)	a*: <0.001, 0.033, <0.001	0	0
2	19 (22.6)	3 (10.7)	23 (29.9)	1 (1)	a*: <0.001, 0.033, <0.001	100 (100)	100 (100)

a*: *P* value 2008 *vs* 2023; 2008/2 *vs* 2023 and 2014 *vs* 2023, respectively
All *P*-value were calculated by Chi square and 2-tailed Fisher's exact tests
Statistically significant difference between years at *P*-value < 0.001.
